# Fructooligosaccharide raftilose reduces the mycophenolate mofetil-induced complications: Hematological and biochemical alterations

**Published:** 2015-12-15

**Authors:** Hadi Cheraghi, Zohreh Khaki, Hassan Malekinejad, Farhang Sasani

**Affiliations:** 1 *Department of Clinical Pathology, Faculty of Veterinary Medicine, University of Tehran, Tehran, Iran;*; 2 *Department of Pharmacology and Toxicology, Faculty of Pharmacy, Urmia University of Medical Sciences, Urmia, Iran;*; 3 *Department of Basic Sciences, Faculty of Veterinary Medicine, Urmia University, Urmia, Iran;*; 4 *Department of Veterinary Pathology, Faculty of Veterinary Medicine, University of Tehran, Tehran, Iran.*

**Keywords:** Fructooligosaccharide, Hematology, Mycophenolate mofetil, Oxidative stress

## Abstract

Mycophenolate mofetil (MMF) is a selective inhibitor of Inosine-5′-monophosphate dehydrogenase. Gastrointestinal (GI) disturbances in immature ones are reported for MMF-induced compilations, which in the case of occurrence dose reduction is required. Thus, in the present study, the fructooligosaccharide raftilose^®^ (RFT) was co-administrated with MMF to estimate the protective effect of RFT against MMF-induced GI complications. Thirty six immature male Wistar rats were divided into six groups including: Control (normal saline), RFT-treated (100 mg kg^-1^), MMF-treated (20 mg kg^-1^), MMF + LRFT (50 mg kg^-1^), MMF + MRFT (100 mg kg^-1^) and MMF + HRFT (200 mg kg^-1^) groups. The hematocrit (Hct), lymphocyte/total WBC, feces water content and pH were analyzed. Moreover, the hepatic functional tests, kidney-related biomarkers, lipid and protein profiles, total antioxidant capacity (TAC), malondialdehyde (MDA) and nitric oxide (NO) contents were assessed. Co-administration of RFT stabilized the MMF-reduced body weight. The MMF significantly diminished Hct and lymph/total WBC (*p* < 0.05). Only MRFT enhanced the lymphocyte/total WBC. Increased water content, no changes in feces pH, increased serum ALT and AST, no alteration in urea and mild enhancement in creatinine were demonstrated in MMF-received animals. However, RFT at low dose ameliorated the feces parameters and reduced ALT. No significant changes were demonstrated for serum lipid and protein profiles in MMF- and RFT + MMF-treated groups. The RFT enhanced the serum TAC, reduced MDA and NO contents. In conclusion, our data suggested that RFT could be considered as an effective agent to subsidize the MMF-induced clinical, hematological and biochemical disorders.

## Introduction

Mycophenolate mofetil (CellCept^®^), as an immune-suppressant agent, is currently used for post-transplantation care and immune-mediated diseases.^1^^,^^2^ Mycophenolate mofetil (MMF), a pro-drug of myco-phenolic acid, is a potent inhibitor of the type II isoform of Inosine-5′-monophosphate dehydrogenase (IMPDH), which is expressed in activated lymphocytes. IMPDH is known as an essential enzyme in the *de novo* purine synthesis pathway. Due to dominant expression of IMPDH in lymphocytes (B and T Types), it is considered as an important cytostatic enzyme in these cells.^3^^,^^4^ Moreover, MMF in comparison with other widely used immune-suppresors potently inhibits the lymphocytes proliferation and glycosylation as well as expression of adhesion molecules. Therefore, it has been known as an appropriate/selective compound for inhibiting the type II IMPDH isoform expression, explaining its beneficial effects in the reduction of organ rejection.^5^

Although MMF is widely used in post-transplantation therapies, there are several reports indicating that long-time administration of MMF results in gastrointestinal (GI) side effects including: typical diarrhea, bloating, nausea, abdominal pains and bacterial infections in approximately 45% of patients.^6^ All these complications result in dose reduction and/or discontinuing the drug administration which elevate the risk of rejection and/or infections.^7^ Severe forms of GI intolerance are reported in immature patients because of their lack of development in entero-hepatic circulation, hence, selection of a suitable dose as well as severity of dose-depending side effects, are known to be more notable in pediatrics.^8^

Prebiotics are simple and non-digestible ingredients that improve host health, stimulating growth and activity of beneficial bacteria in the GI tract. It has been recently found that prebiotics play important role in reducing period of diarrhea and are widely used to diminish damages associated with Crohn’s disease and ulcerative colitis.^9^ The principal characteristics of a prebiotic are resistance to digestive enzymes in the GI but fermentability by the colonic microflora and pH-lowering effects. Also, prebiotics could improve the intestinal barrier by stimulating the growth of protective bacteria such as *Bifidobacteria* and *Lactobacillus*, which provoke epithelial protective mechanisms against intestinal inflammation in animal models of colitis.^10^ On the other hand, they could restore intestinal epithelial integrity by enhancing tight junctions and increasing mucus production.^11^

An important prebiotic which is present in many edible fruits and vegetables is fructooligosaccharide (FOS). Raftilose^®^ P95 (RFT) is a commercial fructooligosaccharide, which is water soluble without any effect on intestinal content viscosity.^12^ Due to special chemical structure, it is not subjected to absorption in small intestine, however, it is fermented in large intestine. End products of the fermentation with endogenous bacteria are lactic and short chain carboxylic acid which control intraluminal pH. Some studies suggest that an increase in *bifidobacteria* and *lactobacillus* in the GI tract after using prebiotics, decreases inflammatory cytokines; likewise neutralizes bacterial toxins and improve intestinal barrier.^13^^,^^14^

In this study, we aimed to investigate the possible beneficial effects of prebiotic RFT on the attenuation of MMF-induced GI disturbances. The MMF-induced oxidative and nitrosative stress plus inflammation were taken in account and the ameliorative effect of RFT was evaluated.

## Materials and Methods

**Chemicals. **Mycophenolate mofetil was purchased from Hoffman La Roche (Basel, Switzerland). 5.5’-dithiobis-2-nitrobenzoic acid (DTNB), N-(1-naphthyl) ethylenediamine dihydrochloride (NED), hexadecyl-trimethyl ammonium bromide, and tetramethylbenzidine NED were obtained from Sigma-Aldrich (Schnelldorf, Germany). Thiobarbituric acid, phosphoric acid (85%), dimethyl sulfoxide (DMSO), sodium nitrite and ethanol were purchased from Merck (Darmstadt, Germany). N-butanol was obtained from Carl Roth, GmbH Co. (Karlsruhe, Germany). Sulfanilamide was purchased from ACROS (Acros Chemical, Princeton, USA). All other chemicals were commercial products of analytical grade. 

**Animals and experimental design. **This study was carried out on 36 immature male Wistar rats, aged 4 weeks, weighing approximately 30 to 50 g. The rats were acclimatized for approximately one week before use and placed in plastic cages with *ad labium* access to standard chow and tap water. They were kept under a controlled room temperature (20 ± 2 ˚C) with a constant 12-hr/12-hr light/dark cycle that was approved by the Institutional Animal Care Committee. The rats were divided into six groups of six animals each, as follow: 

Control group (C): rats in this group only received normal saline; 

Mycophenolate mofetil group: rats in this group received 20 mg kg^-1 ^MMF orally; 

Raftilose group: animals in this group received 100 mg kg^-1^ RFT orally. 

The last three groups in addition of 20 mg kg^-1 ^MMF, were treated with various levels of RFT (50, 100 and 200 mg kg^-1 ^and nominated as LRFT, MRFT and HRFT, respectively) orally. The RFT and saline were administered every day at 9:00 am and MMF administration was performed at 15:00 pm to minimize any possible drug-drug interactions.

All rats were examined on a daily basis for clinical signs of diarrhea (loose, watery, and frequent stools) and were weighed weekly. At the end of the study period (28 days), animals were weighed and then anesthetized using diethyl ether and blood samples were taken from the heart to determine the hematological and biochemical factors.

**Water content and pH of GI contents. **At the end of the study period, before euthanizing the rats, content of digestive tract was collected, weighed and dried at 80 ˚C in an oven for 24 hr, and then reweighed. Water content was calculated from subtraction of the fecal wet weight from the dry weight. For determination of pH, 100 mg of content was freshly collected and homogenized with 2 mL of 9 g L^-1^ NaCl and then pH was immediately determined with digital pH meter. 

**Hematology analysis. **Hematological parameters including, hematocrit value (Hct), total white blood cells (WBC) and differential leukocyte counts were determined manually as described by Meyer and Harvey.^15^ Also, lymphocyte to total WBC ratio was evaluated to show effect of MMF on this ratio.

**Biochemical parameters. **For testing hepatic and renal function, the serum level of liver functional enzymes including alanine aminotransferase (ALT) and aspartate aminotransferase (AST) as well as urea and creatinine were assessed using an auto-analyzer (EliTech Diagnostic, Sées, France) based on the manufacturer’s instructions. 

To determine lipid, protein profile and glucose level in serum, the lipid profile, cholesterol and triglyceride levels in serum of all groups were measured. Other biochemical parameters such as total protein, albumin and glucose levels were also determined using the auto-analyzer and commercial kits (EliTech Diagnostic, Sées, France). Also, globulin concentration was calculated subtracting the serum albumin from the total protein concentration.^16^

**Total antioxidant capacity (TAC). **The ferric reducing/ antioxidant power (FRAP) assay was performed to measure the total antioxidant capacity in serum as previously described in detail.^17^

**Nitric oxide (NO) assay. **The total NO content of the sera was measured according to the Griess reaction which is previously described in detail.^18^ In the Griess reaction, NO is rapidly converted into the more stable nitrite, which in an acidic environment is converted into HNO_2_. In reaction with sulphanilamide, HNO_2_ forms a diazonium salt, which reacts with N-(1-Naphthyl) ethylenediamine dihydrochloride to form an azo dye that can be detected by the absorbance at a wavelength of 540 nm. The NO content of the examined organs was expressed as nmol per mg of protein in samples.

**Malondialdehyde (MDA) assay. **To determine the lipid peroxidation rate, MDA content of the collected tissue samples was measured using the TBA reaction as described previously.^19^ In brief, 0.5 mL of the serum samples was mixed with 3 mL phosphoric acid (1% v/v) and then following vortex mixing, 1 mL of 6.7 g L^-1^ TBA was added to the samples. The samples were heated at 100 ˚C for 45 min and chilled on ice. Finally, 3 mL N-butanol was added and the samples were further centrifuged at 3000 rpm for 10 min. The absorbance of supernatant was measured spectrophotometerically at 532 nm and the concentration of MDA was calculated according to the simultaneously prepared calibration curves using MDA standards. The amount of MDA was expressed as nmol per mg protein of the samples. The protein content of the samples was measured according to Lowry’s method.^20^

**Statistical Analysis. **Statistical mean and standard deviation of the values were measured. The results were analyzed using Graph Pad Prism software (version 6.01, Graph Pad software Inc. San Diego, USA). The comparisons between groups were made by analysis of variance (ANOVA) followed by Bonferroni post*-*hoc test. A *p*-value less than 0.05 was considered statistically different.

## Results

**Fig. 1:**
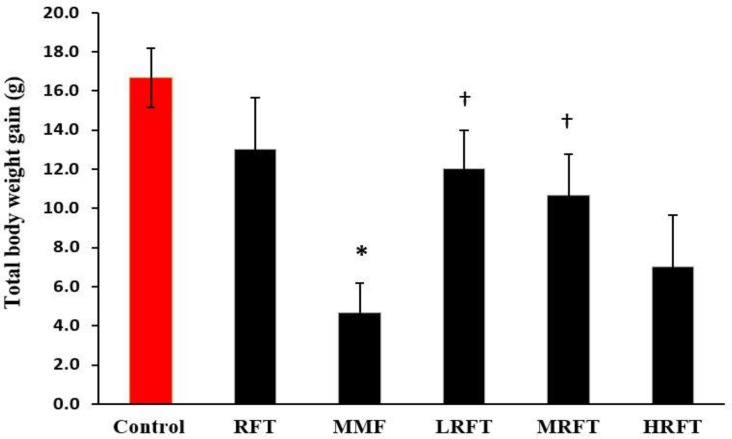
Effects of mycophenolate mofetil (MMF) and raftilose (RFT) on total body weight gain. RFT-treated (100 mg kg^-1^), MMF-treated (20 mg kg^-1^), MMF + LRFT (50 mg kg^-1^), MMF + MRFT (100 mg kg^-1^) and MMF + HRFT (200 mg kg^-1^). Data are shown as mean ± SD.

**General findings. **Severe diarrhea was observed in MMF-received animals on day seven after first administration. In contrast, no diarrhea was recorded in the low and medium dose RFT-treated groups. Interestingly, the high dose administration of RFT resulted in diarrhea two weeks later. Moreover, the total body weight (TBW) gain in all groups was evaluated at the end of experiment period. Observations demonstrated that MMF significantly (*p *< 0.05) reduced the TBW versus the control and other test groups. However, RFT-treated animals exhibited remarkably (*p *< 0.05) higher TBW in comparison with MMF-received group. Comparing LRFT, MRFT and HRFT groups with MMF group, revealed that RFT at low and medium dose levels significantly (*p *< 0.05) elevated the TBW. Meanwhile, the high dose-received group showed no significant (*p *> 0.05) alterations in TBW (Fig. 1).

* represents a significant difference between MMF-received and control group (*p* < 0.05); ^†^ shows significant differences between MMF-received untreated and RFT-treated groups (*p* < 0.05).

**Fecal analysis.** Table 1 presents the alterations of fecal water content and pH values in different groups. MMF enhanced the water content compared to the control group. Comparing the water content of all other groups with MMF-received animals showed a remarkable reduction in water content in LRFT and MRFT. Meanwhile, no significant differences were demonstrated for pH between MMF- and RFT-received groups in comparison with control group. However, the pH values were remarkably increased in LRFT and significantly decreased in HRFT group in comparison with MMF-received animals. 

**Fig. 2:**
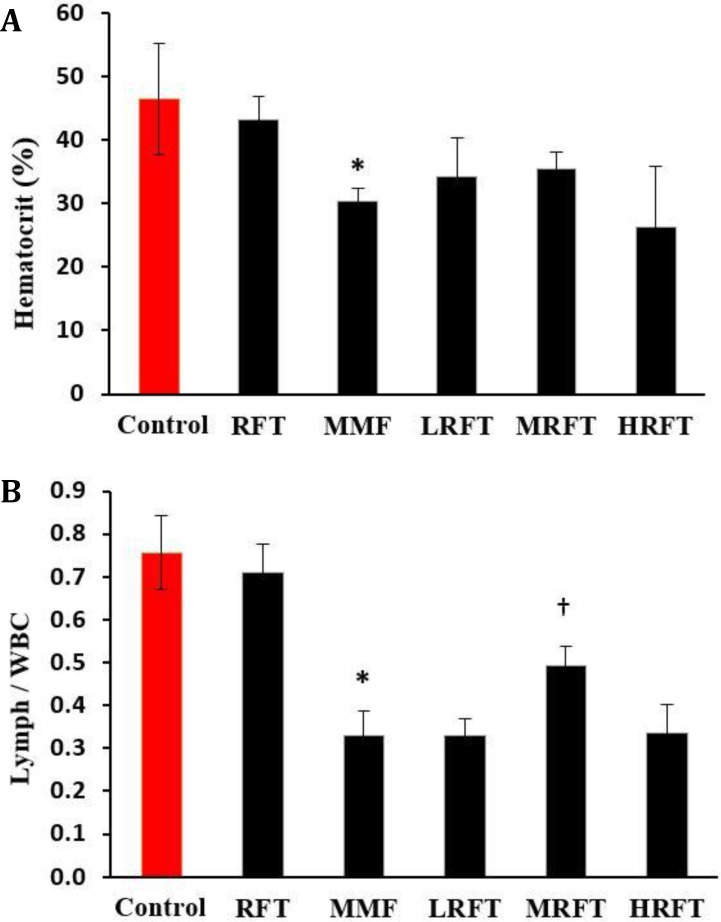
Effects of mycophenolate mofetil (MMF) and raftilose (RFT) on: **A)** Hematocrit values in all experimental groups, and **B)** Lymphocyte to total white blood cells ratio. Data are shown as mean ± SD. RFT-treated (100 mg kg^-1^), MMF-treated (20 mg kg^-1^), MMF + LRFT (50 mg kg^-1^), MMF + MRFT (100 mg kg^-1^) and MMF + HRFT (200 mg kg^-1^).

**Hematological findings. **Hematological examinations showed that the percentage of Hct was decreased in the MMF-received group versus control and RFT-received animals. The lowest values of Hct were seen in the HRFT group but no significant differences were revealed between all MMF-received animals in comparison with MMF-received group (Fig. 2A). The MMF-received animals exhibited a significant reduction in the lymphocyte to total WBC ratio compared to the control group. In comparison between the RFT-received and MMF-treated groups, only MRFT-received animals showed statistical differences (Fig. 2B). 

**Hepatic and renal functional analysis. **The serum levels of AST and ALT were analyzed in order to evaluate the liver function parameters. MMF-received animals exhibited remarkable enhancement in serum levels of AST and ALT versus the control animals, whereas RFT reduced the serum level of AST (Table 2). The serum levels of urea and creatinine were analyzed in order to evaluate the kidney function. Observations showed no significant changes in serum level of urea in all groups, while the creatinine level was significantly elevated in the MMF-treated animals in comparison with the control group (Table 2).

**Lipid, protein profile and glucose level analysis. **The cholesterol and triglyceride levels of serum were assessed as special markers for lipid profiles. In comparison with the control group, serum level of cholesterol was diminished in RFT-received group. The highest level of cholesterol was seen in MMF-received group. Meanwhile, administration of MRFT and HRFT significantly (*p* < 0.05) reduced the MMF-increased serum level of cholesterol. On the other hand, MMF did not exert any significant alteration in serum level of triglyceride, however, RFT in individual form of administration remarkably (*p* < 0.05) reduced the triglyceride content when compared to the control group. Interestingly, the serum concentration of triglyceride significantly (*p* < 0.05) was increased in LRFT group and contrarily was decreased in HRFT group (Table 3). 

Observations demonstrated no significant changes in the total protein, albumin and globulin of the serum in MMF-and RFT-treated groups. Comparing the co-treated animals with each other showed that except for the LRFT-received animals other co-treated animals showed remarkable (*p* < 0.05) reduction in serum total protein content. However, no significant changes were revealed for albumin and globulin levels in all co-treated animals (Table 3).

Finally, the serum level of glucose was assessed and biochemical results illustrated that both RFT and MMF in single form of administration enhanced the glucose level. All groups except for HRFT group showed an enhanced glucose level compared to the control (Table 3). 

* represents a significant difference between MMF-received and control group (*p* < 0.05); ^†^ shows significant differences between MMF-received untreated and RFT-treated groups (*p* < 0.05).

**Table 1:** Effects of mycophenolate mofetil (MMF) and raftilose (RFT) on fecal water content and pH values. Data are shown as mean ± SD. RFT-treated (100 mg kg^-1^), MMF-treated (20 mg kg^-1^), MMF + LRFT (50 mg kg^-1^), MMF + MRFT (100 mg kg^-1^) and MMF + HRFT (200 mg kg^-1^).

**Parameters**	**Control**	**RFT**	**MMF**	**LRFT **	**MRFT **	**HRFT **
**Water content**	1.30 ± 0.52	2.12 ± 1.19	2.20 ± 0.11*	0.42 ± 0.05†	1.85 ± 0.04†	3.21 ± 1.15
**pH**	6.20 ± 0.04	6.30 ± 0.06	6.15 ± 0.09	6.26 ± 0.04†	6.07 ± 0.03	5.89 ± 0.01†

* represents a significant difference between MMF-received and control group;

† shows significant differences between MMF-received untreated and RFT-treated groups (*p* < 0.05).

**Table 2:** Effects of mycophenolate mofetil (MMF) and raftilose (RFT) on hepatic functional enzymes and renal functional bio-markers. Data are shown as mean ± SD. RFT-treated (100 mg kg^-1^), MMF-treated (20 mg kg^-1^), MMF + LRFT (50 mg kg^-1^), MMF + MRFT (100 mg kg^-1^) and MMF + HRFT (200 mg kg^-1^

**Groups**	**ALT** **(U L** ^-1^ **)**	**AST** **(U L** ^-1^ **)**	**Urea** **(mg dL** ^-1^ **)**	**Creatinine** **(mg dL** ^-1^ **)**
**Control**	16.33 ± 4.50	56.33 ± 6.50	34.67 ± 1.50	0.87 ± 0.06
**RFT**	18.50 ± 1.50	35.67 ± 9.20*	43.00 ± 10.30	0.70 ± 0.17
**MMF**	37.33 ± 5.30*	69.20 ± 2.30*	43.67 ± 8.70	1.27 ± 0.32*
**LRFT **	24.67 ± 5.70†	69.00 ± 4.90	55.33 ± 3.50	0.93 ± 0.06
**MRFT **	37.00 ± 6.30	83.50 ± 6.10†	63.67 ± 17.80	1.17 ± 0.25
**HRFT **	35.20 ± 5.20	79.33 ± 3.60†	35.67 ± 5.50	1.07 ± 0.57

ALT = Alanine aminotransferase, AST = Aspartate aminotransferase.

* represents a significant difference between MMF-received and control group (*p* < 0.05);

† shows significant differences between MMF-received untreated and RFT-treated groups (*p* < 0.05).

**Fig. 3:**
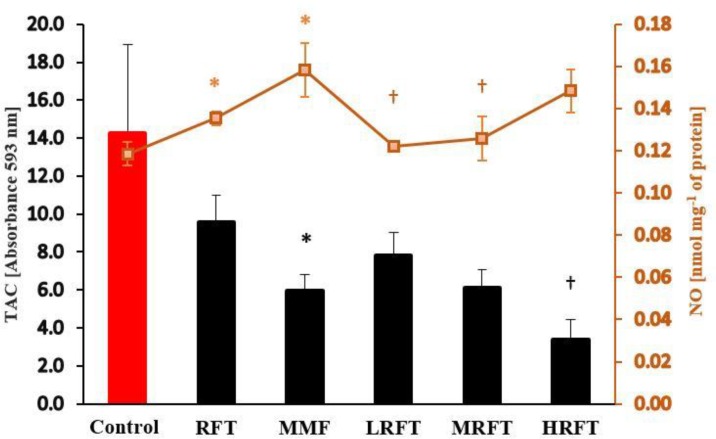
Effects of mycophenolate mofetil (MMF) and raftilose (RFT) on nitric oxide (NO) level and total antioxidant capacity (TAC) in serum. Data are shown as mean ± SD. RFT-treated (100 mg kg^-1^), MMF-treated (20 mg kg^-1^), MMF + LRFT (50 mg kg^-1^), MMF + MRFT (100 mg kg^-1^) and MMF + HRFT (200 mg kg^-1^).

**Fig. 4:**
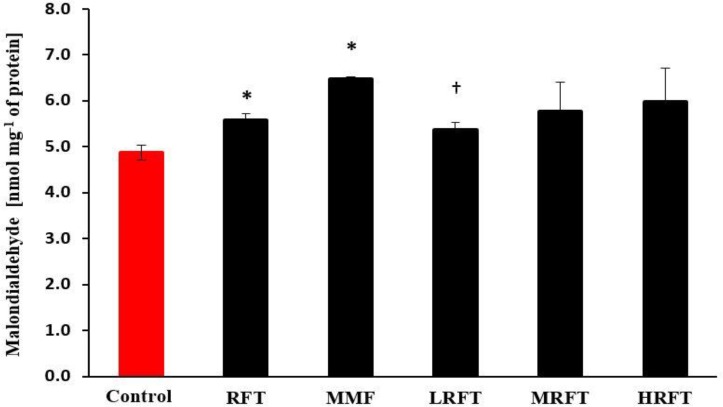
Effects of mycophenolate mofetil (MMF) and raftilose (RFT) on lipid peroxidation rate in serum. Data are shown as mean ± SD. RFT-treated (100 mg kg^-1^), MMF-treated (20 mg kg^-1^), MMF + LRFT (50 mg kg^-1^), MMF + MRFT (100 mg kg^-1^) and MMF + HRFT (200 mg kg^-1^).

**Oxidative stress. **The serum TAC and NO content were evaluated in all experimental groups (Fig. 3). At the end of study, the TAC level in MMF-treated group was estimated significantly lower than the control group (*p* < 0.05). Although co-administration of RFT with MMF elevated the TAC in LRFT and MRFT groups compared to MMF-received group, however, no significant differences were observed between them (*p* > 0.05). The notable point was that co-administration of HRFT led to intensive decrease in serum TAC level. On the other hand, results showed that both MMF and RFT increased the serum level of NO in comparison with control group. However, in comparison between all MMF-received groups, serum level of NO was significantly (*p* < 0.05) diminished in LRFT and MRFT co-treated groups. The serum content of MDA was estimated as a biomarker for lipid peroxidation rate as well as oxidative stress. Observations illustrated that similar to NO the RFT and MMF in single form of administration elevated the MDA content compared to the control group. In contrast to NO content, both low and medium dose levels of RFT could lower the MMF-induced NO increase, and MDA level was reduced only at low dose of RFT (Fig. 4).

* represents a significant difference between MMF-received and control group (*p* < 0.05); ^†^ shows significant differences between MMF-received untreated and RFT-treated groups (*p* < 0.05).

* represents a significant difference between MMF-received and control group (*p* < 0.05); ^†^ shows significant differences between MMF-received untreated and RFT-treated groups (*p* < 0.05).

**Table 3:** Effects of mycophenolate mofetil (MMF) and raftilose (RFT) on hepatic functional enzymes and renal functional bio-markers. Data are shown as mean ± SD. RFT-treated (100 mg kg^-1^), MMF-treated (20 mg kg^-1^), MMF + LRFT (50 mg kg^-1^), MMF + MRFT (100 mg kg^-1^) and MMF + HRFT (200 mg kg^-1^).

**Groups**	**Cholesterol** **(mg dL** ^-1^ **)**	**Triglyceride** **(mg dL** ^-1^ **)**	**Total protein** **(mg dL** ^-1^ **)**	**Albumin** **(mg dL** ^-1^ **)**	**Globulin** **(mg dL** ^-1^ **)**	**Glucose** **(mg dL** ^-1^ **)**
**Control**	59.33 ± 4.20	72.00 ± 20.10	7.29 ± 0.40	3.39 ± 0.30	3.90 ± .023	100.00 ± 18.20
**RFT**	35.67 ± 8.10*	39.67 ± 8.60*	6.87 ± 0.80	2.86 ± 0.60	4.01 ± 0.65	269.33 ± 18.20*
**MMF**	70.00 ± 14.00	64.00 ± 30.80	7.79 ± 0.20	3.00 ± 0.90	4.79 ± 0.98	265.67 ± 21.70*
**LRFT **	65.33 ± 3.50	96.67 ± 33.50†	7.22 ± 1.10	2.79 ± 0.50	4.43 ± 1.59	232.00 ± 28.80
**MRFT **	48.33 ± 4.90†	65.00 ± 4.00	7.07 ± 0.20†	3.53 ± 0.30	3.54 ± 0.16	216.67 ± 52.30
**HRFT **	42.67 ± 7.20†	50.33 ± 4.50†	5.64 ± 0.50†	2.96 ± 0.20	2.65 ± 0.37	120.33 ± 54.00†

* represents a significant difference between MMF-received and control group (*p* < 0.05);

† shows significant differences between MMF-received untreated and RFT-treated groups (*p* < 0.05).

## Discussion

Our findings showed that RFT at medium tested dose level could reflect its ameliorative effects on the MMF-induced injuries including clinical, biochemical, hemato-logical, oxidative stress and fecal assessment. The results of daily-based examinations of the animals in all test groups showed that MMF-treated animals demonstrated a marked loss of body weight along with diarrhea, which time-dependently was worsened. We and others in previous studies showed that MMF with severe villous atrophy results in mal-absorption and consequently weight loss.^18^^,^^21^^,^^22^ One may note the fact that the observed diarrhea also is explainable with the abovementioned injuries to villi. The time-dependent effect of MMF on body weight loss and the severity of observed diarrhea could be interpreted by progressive in villi injuries.^22^

A significant decline of hematocrit level was obtained in the MMF-treated animals when compared to the RFT-received and control groups. One of the hematological toxicity of immunosuppressive agents and in particular MMF is the bone marrow suppression. Mycophenolic acid as the active substance of MMF suppresses *de novo* purine and nucleic acid synthesis via reversibly and non-competitively antagonizing IMPDH activity of bone marrow cells.^23^ There is another report from a kidney transplant recipient with pure red cell aplasia, which discontinuation of immunosuppressive MMF resulted in resolution of mentioned red cell aplasia, confirming and supporting our findings about the hematocrit reduction in MMF-received animals.^24^ Tjeertes *et al*. reported that MMF-administration in mother resulted in neonatal anemia and hydrops fetalis. The authors concluded that the MMF administration in pregnant mother resulted in bone marrow suppression which in turn caused a fetal anemia and consequently non-immune hydrops fetalis.^25^

A significant increase of water content of feces collected from the MMF-received animals confirmed our daily recorded diarrhea. We recently and others previously showed that one of the histological changes in the MMF-treated cases was villous atrophy, which in turn due to mal-absorption could be resulted in diarrhea.^18^^,^^22^^,^^26^ We also recorded a slight and non-significant reduction of pH in the collected feces samples from the MMF-alone treated animals in comparison with control group. The possible reason for this non-significant changes could be the conversion of pro-drug MMF into its active form of mycophenolic acid that may alter slightly the pH level of intestinal content. 

Hematological analyses showed the animals that received medium dose of RFT had the lowest changes in the lymphocyte to total white blood cells ratio, compared to the control and RFT-received animals. This ratio was dramatically declined in the MMF-received animals, suggesting considerable dependency of lymphocyte proliferation on the *de novo* synthesis of guanosine nucleotides than other cell types.^27^ It has been documented that mycophenolic acid as the active substance of MMF provides effective immunosuppression by inhibiting inosine monophosphate dehydrogenase, which acts as a key enzyme in the lymphocytes proliferation.^28^

Biochemical analyses of the serum from MMF-treated group showed no significant alterations in the lipid profile in comparison with control group. The serum level of glucose however showed a marked elevation when it was compared to the control group. To explain the elevated glucose level in the MMF-treated animals, one should note that undoubtedly inflammatory reactions due to the MMF-induced GI injuries are inevitable as it has been reported previously.^21^^,^^29^ Serum level of nitric oxide and the end product of lipid peroxidation (*i.e.* MDA) along with total antioxidant capacity were assessed to indicate any changes in antioxidant power in the MMF-received animals. Results showed that both NO and MDA levels significantly were enhanced in the serum of MMF-treated animals, while antioxidant capacity was remarkably reduced, suggesting a pro-oxidant effect of the MMF administration. There are some previous data, which do not support our findings. For example, Dalmarco *et al*. reported that MMF not only reduced the leukocyte influx but also declined the lipid peroxidation levels 4 and 48 hr after carrageenan-induced pleural cavity inflammation.^30^ Another study also reported that MMF administration suppressed the tubule interstitial accumulation of lympho-cytes and macrophages and consequently declined the lead-induced oxidative stress in the kidneys.^31^ To explain this controversy, it should be noted the organ of study, duration of MMF administration, whether the antioxidant capacity were measured locally or systematically and more importantly how long after the MMF administration the evaluations were performed. The well documented entro-colitis induced by the MMF administration was mentioned above and the obtained results of the current study with clinical symptoms including severe diarrhea confirmed GI injuries due to MMF administration. Therefore, our findings indicated that MMF administration for relatively long time with pro-oxidative properties such as increased MDA and NO levels may result in declining of total antioxidant capacity.

The second part of the present study was devoted to explore any beneficial effects of RFT on MMF-induced disorders. We found that RFT in low and medium and not high given dose level could protect from MMF induced injuries. It should be noticed that both MMF and RFT have been co-administered, therefore, the protective effects of RFT was the aim of co-administration. To explain that how and based on which mechanism RFT at low and medium given dose levels could protect from detrimental effects of MMF, one should bear in mind the following approaches which are related to prebiotics: (i) RFT with binding to MMF reduces its plasma concentration, (ii) RFT with modifying the GI microflora gives an opportunity to replace target genera from bacteria including *bifidobacteria* and *lactobacilli*, which in turn may convert or degrade the MMF and/or inactivate it, (iii) RFT provokes immuno-logical reactions in enterocytes to be stimulated and alter the signal transduction pathways. Our results confirmed that at low and medium tested doses RFT plays a protective role. We already discussed that the main reason of MMF-induced diarrhea could be villous atrophy and mal-absorption along with mal-digestion. In this regard, it has been well documented that prebiotics including RFT with changing the GI microflora and replacing some beneficial bacteria not only improve the digestion and absorption processes but also improve the enterocytes integrity and their functions. For example, it has been reported that some *lactobacilli* stimulate the production of mucus in the intestinal tract, which may contribute in the protecting from injuries including MMF-induced damages.^32^ Another beneficial effect of prebiotics like RFT may be explained by alteration of GI tract bacterial density, as an increase in the density of *lactobacilli* and *bifidobacteria* likely prevents the proliferation of pathogenic bacteria.^33^

Previous studies showed that in particular gram negative bacteria such as *E. coli* via activation of TLR4 in GI resulted in a high production of superoxide and increase of nuclear factor-kappa B (NF-κB) activity and pro-inflammatory factors, which ultimately cause an inflammation and local and systemic oxidative stress.^34^ We showed that 28 days co-treatment with RFT resulted in a remarkable reduction of lipid peroxidation and significant enhancement of total antioxidant capacity in MMF-received rats. There are supporting data which indicating antioxidant and anti-inflammatory and anti-obesity effects of prebiotics via altered intestinal microbial composition.^35^

Our results did not show dose-dependent antioxidant and anti-diarrhea effects. We witnessed a severe diarrhea and oxidative stress at the highest given dose of RFT along with MMF. An acceptable reason for these finding could be strong osmotic environment which has been developed when the high dose of RFT was administered for relatively long period of time. The observed weight loss and marked reduction of intestinal content pH along with dramatically increased water content are indicating an osmotic cathartic property of RFT at high dose level. 

In conclusion, our data showed that prebiotic RFT could be considered as an effective agent to subsidize the MMF-induced clinical, hematological and biochemical disorders. There is absolute need to uncover the molecular mechanism of action for these findings in detail. 
